# Biogeographical Distribution of River Microbial Communities in Atlantic Catchments

**DOI:** 10.1111/1758-2229.70065

**Published:** 2025-01-08

**Authors:** Alejandra Goldenberg‐Vilar, María Morán‐Luis, David R. Vieites, José Manuel Álvarez‐Martínez, Ana Silió, Cendrine Mony, Simone Varandas, Sandra Mariza Monteiro, Diane Burgess, Edna Cabecinha, José Barquín

**Affiliations:** ^1^ IHCantabria—Instituto de Hidráulica Ambiental de la Universidad de Cantabria Universidad de Cantabria Santander Spain; ^2^ Instituto de Investigaciones Marinas, Consejo Superior de Investigaciones Científicas Vigo Spain; ^3^ Biodiversity Research Institute IMIB (Univ. Oviedo‐CSIC‐Princ. Asturias) Mieres Spain; ^4^ University of Rennes, UMR CNRS Ecobio Rennes Cedex France; ^5^ Centre for the Research and Technology of Agro‐Environmental and Biological Sciences, CITAB/Inov4Agro Universidade de Trás‐Os‐Montes e Alto Douro, UTAD Vila Real Portugal; ^6^ BIOPOLIS Program in Genomics, Biodiversity and Ecosystems Universidade do Porto Vairão Portugal; ^7^ CIBIO Research Centre in Biodiversity and Genetic Resources Universidade do Porto Vairão Portugal; ^8^ InBIO Research Network in Biodiversity and Evolutionary Biology Universidade do Porto Vairão Portugal; ^9^ Agriculture and Food Bioscience Institute Belfast Northern Ireland, UK

**Keywords:** Atlantic landscapes, eDNA, eukaryotes, freshwater microbial communities, land use, prokaryotes

## Abstract

Microbes inhabit virtually all river ecosystems, influencing energy flow and playing a key role in global sustainability and climate change. Yet, there is uncertainty about how various taxonomic groups respond to large‐scale factors in river networks. We analysed microbial community richness and composition across six European Atlantic catchments using environmental DNA sequencing. Our findings reveal different drivers for diversity and composition: land use is pivotal for eukaryotes, while climate and geology are crucial for prokaryotes. A strong regional influence shapes these communities, with warmer, drier regions (Portugal and France) differing from cooler, wetter ones (Northern Spain, Ireland and the United Kingdom). These patterns suggest potential indicators for global change, such as taxa resistant to temperature increases and water scarcity, or those sensitive to land use changes.

## Graphical abstract

We discovered different factors affecting river microbial communities (RMC) diversity and composition across Atlantic catchments in Europe. Prokaryote diversity was mainly influenced by topography and geology, while eukaryotic groups were driven by land use and cover. Species composition showed consistency across taxa, with many of the same environmental factors influencing most groups, indicating a strong regional impact on RMC composition. Our findings highlight distinctions between communities in warmer, drier catchments in Portugal and France versus cooler, wetter areas in Northern Spain, Ireland and the United Kingdom.

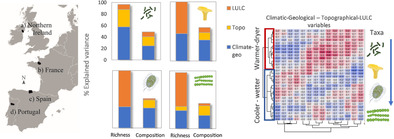

## 1 Introduction

Global biodiversity is significantly threatened by the combined effects of human‐induced climate and land use changes which tend to potentially exacerbate both the pace and the amplitude of the current biodiversity crisis (IPBES 2019). Fluvial ecosystems are particularly vulnerable to climate change and anthropogenic impacts as they integrate all the effects of human activities across the landscapes and river networks (Markovic et al. 2017; David et al. 2021). Among all organisms inhabiting riverine ecosystems, microbes occupy almost all aquatic habitats across environmental gradients (Mansour et al. 2018), encompassing a high diversity (Staley et al. 2015) including prokaryotes (bacteria and archaea), fungi, algae, protozoans and meiobenthos (Veach and Griffiths 2018). They can be free‐living plankton communities inhabiting the water column (David et al. 2020) or biofilms formed in and inhabiting benthic and hyporheic sediments (Valentine and Mariotti 2020). This high diversity is related to major ecosystem functions as they are keystones for biogeochemical cycles (Cavicchioli et al. 2019), the principal decomposers in aquatic ecosystems (Tlili et al. 2017), pollutant degraders and they are also involved in pathogen control (Delgado‐Baquerizo et al. 2020). As a result, changes in microbial biodiversity induced by global change may affect current ecosystem functioning and resilience. Microbial changes also strongly influence the response of other organisms to climate change (Cavicchioli et al. 2019). For example, soil microbial diversity influences plant diversity because of their role in nutrient cycling (Walker et al. 2018). Therefore, improving our understanding of how these communities change in response to climate variability and human activities is essential for defining appropriate management actions to preserve ecosystem multifunctionality despite environmental changes (Delgado‐Baquerizo et al. 2020).

Biodiversity patterns in riverine environments are mostly studied considering only macroorganisms (e.g., macroalgae, macrophytes, macroinvertebrates and vertebrate communities; Metz et al. 2022; Seena et al. 2019), overlooking microorganisms, and especially eukaryotes. Concerning microbial communities, the molecular diversity of microbial eukaryotes in aquatic ecosystems is far less investigated than their prokaryotic counterparts. Moreover, most of the studies dealing with river microbial communities (RMC) focus on a single microbial taxonomic domain (only prokaryotes or only eukaryotes), while only 1% of the studies investigate prokaryotes and eukaryotes simultaneously (Li, Gao, et al. 2021, Li, Hu, et al. 2021). RMC have been described in relationship to water physicochemical properties, such as pH, conductivity, temperature, dissolved oxygen, as well as nitrogen‐related properties and phosphorus‐related indices (Li, Gao, et al. 2021, Li, Hu, et al. 2021), while their response to large‐scale environmental factors has been much less investigated. Large‐scale factors in rivers include climatic, geological, hydrological and topographical catchment characteristics and land use and land cover (LULC) composition. Some of the studies addressing these large‐scale factors on RMC include the effect of temperature in fungal communities (Seena et al. 2019), the amount and timing of precipitation on microbial communities (Zeglin 2015) or the effect of land use change in prokaryotes (Chen et al. 2018; Ji et al. 2022; Zhao et al. 2022; Hermans et al. 2017; Griffiths et al. 2011). Moreover, it should be noted that important changes on RMC have been reported at the river network scale. For example, distinct bacterial composition were found in the transition from tributaries to more downstream reaches because of mass effects and species sorting mechanisms (Crump, Amaral‐Zettler, and Kling 2012; Besemer et al. 2013; Read et al. 2015; Staley et al. 2015; Niño‐García, Ruiz‐González, and Del Giorgio 2016). These changes have also been attributed to changes in elevation, hydrological conditions and nutrient concentrations (Besemer et al. 2013; Savio et al. 2015; Wang et al. 2017; Henson et al. 2018).

This knowledge is crucial to increase our understanding on how microbial diversity and activity that governs small‐scale interactions translate to large system fluxes (Delgado‐Baquerizo et al. 2020). Nevertheless, it is still unknown if general patterns can be detected across multiple catchments along large‐scale gradients and how these environmental characteristics will interact to shape RMC diversity and composition. To provide a systematic assessment of the forces driving microbial diversity and composition across large‐scale gradients, the present study aims at examining the response of RMC to biogeographical gradients over human‐dominated catchments at the European scale. We will analyse the effect of regional drivers (climate, geology and hydrology), catchment characteristics (elevation, catchment area) and LULC composition on RMC. The study includes prokaryotes, fungi, protists and algae from both water and biofilm compartments. The inclusion of taxa from multiple kingdoms is critical for supporting the development of robust indicators of ecosystem health and resilience (Battin et al. 2016). More specifically, we aimed at testing the following two main hypotheses: (i) distinct RMC compositional and richness patterns can be detected across catchments likely because climate, geological and hydrological characteristics are different among them; (ii) there is a strong effect of river network position within the entire catchment (i.e., headwaters, middle and lower river reaches) due to changes in physicochemical characteristics and LULC patterns along the river continuum.

## 2 Materials and Methods

### 2.1 Study Sites

**FIGURE 1:**
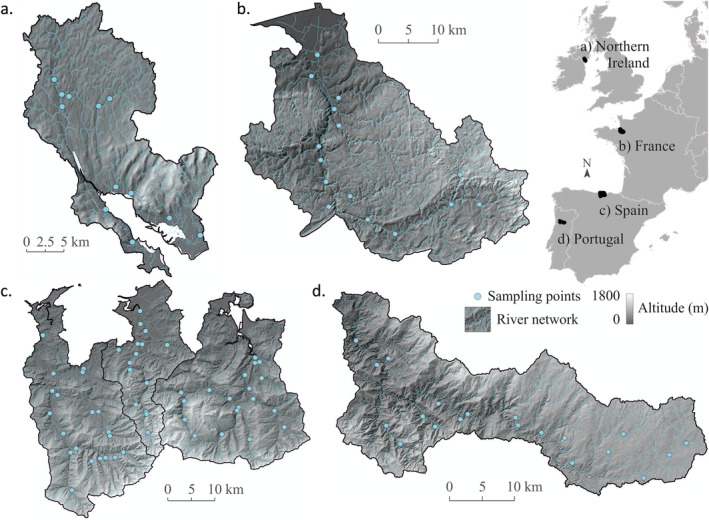
Map of river networks, study area boundaries and sampling sites along the Atlantic catchments: (a) Carlingford Lough (Ireland and the United Kingdom), (b) Couesnon (France), (c) Pas, Miera and Asón (Spain) and (d) Paiva (Portugal).

This study included six catchments distributed across five European countries: Ireland, the United Kingdom, France, Spain and Portugal (Figure 1). The Couesnon River, located in northwestern France, has an 89 km length, and it drains the Armorican massif (1128 km^2^), discharging into the Bay of Mont‐Saint‐Michel (UNESCO World Heritage site). This river presents a relatively smooth orography, with a moderate top elevation (256 m) at the source. The Couesnon River catchment consists mainly of agricultural land with mixed grazing pastures in the upper stream and polders at the interface with the sea (79.6%), followed by artificial areas (7.5%) and a very reduced forest cover (3.6%) (Fonseca et al. 2022). The climate is maritime, with an average monthly temperature ranging between 17.5°C in July and 5°C in December, along with a mean annual precipitation of 787 mm, of which a third occurs from October to December. The low soil permeability (grain and schist rocks) and the important rainfalls cause a dense hydrographic network in the area.

The Carlingford Lough catchment is located on the East Coast of Ireland, across the border between the Republic of Ireland and the United Kingdom. The Lough is a coastal embayment extending 16.5 km into the Irish Sea. The Mourne Mountains in Northern Ireland delimit the catchment in its northern part and the Cooley Mountains in the Republic of Ireland on its southern side. The catchment's highest elevation is 600 m above the Irish Sea. Inflowing catchments to the lough drain an area of 475 km^2^, with the majority lying within the United Kingdom (426 km^2^). The LULC is mainly comprised of meadows (58.8%) and shrubland (15.4%), followed by wetlands (10%), artificial land (5.6%) and forests (5.5%) (Fonseca et al. 2022). The climate in the Carlingford—Lough catchment is temperate, with average monthly temperature ranging from 17°C to 7°C, and average rainfall between 800 and 1000 mm. A combination of metamorphosed shale, sandstones and igneous granites underlies the area.

The Paiva River catchment (796 km^2^), located in northern Portugal, is a medium‐sized watercourse (115 km length) draining into the highly regulated Douro River. The catchment orography is complex, with the river source located approximately 1000 m high and the river junction to the Douro at 10 m high above sea level. This catchment comprises both gentle and very steep slopes, mainly covered by forest, which are mainly eucalyptus plantations (41%) and pasture (38%), followed by agricultural fields (13%) (Fonseca et al. 2022). Most of its catchment is subject to a temperate climate; however, the estuary has a Mediterranean climate with an average monthly temperature ranging from 28°C in August to 11°C in January, and a mean annual precipitation of 977 mm with a marked dry season lasting almost 4 months (from June to September).

The Spanish site includes three neighbouring catchments (Pas, Miera and Asón rivers) draining into the Cantabrian Sea with a total area of 2047 km^2^ and a river length of 223.9 km. The orography is complex, with relatively soft slopes in coastal areas, very mountainous middle internal valleys (elevations from 100 to 1200 m) and upper mountainous ranges with altitudes exceeding 1700 m and very pronounced slopes. Vegetation comprises shrublands (28.2%), meadows (26%), natural forests (21%) and estuarine wetlands (16.7%), with a small proportion of artificial areas (Fonseca et al. 2022). There are also very contrasted LULC patterns across the three catchments, with a historical degradation of natural forests in Pas and Miera while Asón preserves extensive areas of natural forest stands (Belmar et al. 2018). The climate is temperate hyper‐oceanic, with mild temperatures and high humidity due to regular precipitation and fog. Average temperature ranges between 25°C in August and 10°C in February, along with a mean annual precipitation of 1042 mm, but with a high spatial gradient (e.g., over 2000 mm in the mountain tops and below 1000 mm in some coastal areas).

### 2.2 Field Survey

Water and biofilm samples were taken for 103 sites located along the river network to capture within‐catchment variability (15 for Carlingford Lough, Couesnon and Miera; 19 for Asón and Paiva; and 20 for the Pas river). Samples were collected from July to September 2018. Water samples were collected on sterile bottles at intervals of 12 min over 1 h (five bottles of 1 L each). After the collection of the five bottles, one composite sample was created combining 200 mL of each water bottle to a final volume of 1 L, in this way, we created an integrated sample to capture stochastic temporal variability in each river sampling site. The litre was filtered using a sterile filter column with a cellulose nitrate filter (Whatman plc, Maidstone, UK; ø 25 mm, pore size 0.45 μm). While we are aware that some bacterial taxa may be lost using a filter of 0.45, other phyla such as Cyanobacteria, Desulfobacterota, Firmicutes, Gemmatimonadota, Myxococcota and Nitrospirota might be underrepresented, as Byappanahalli et al. 2021 stated. Due this lack of consensus, we chose one of the most standard filter pore size (0.45 μm) for our study in order to capture most of the targeted organisms (prokaryotes and eukaryotes). To characterise benthic biofilms, we selected six cobbles from riffle areas, concentrating on the river's main flow. Zones of slow current (approx. ≤ 20 cm s^−1^) were avoided as they allow the build‐up of loosely attached diatoms, silt and other debris. Cobbles were collected at a depth of ca. 10 cm to ensure that they were not exposed to air in the previous 4 weeks. Areas of heavy shade and those close to the bank were avoided. Stones were thoroughly scrubbed with a sterile dishwasher brush. Around 10 mL of the dislodged material was filtered using a filter with the same characteristics as the one used for water samples. Water and biofilm samples were preserved in absolute ethanol and stored at −22°C right after being surveyed.

### 2.3 DNA Extraction, PCR Amplification, Sequencing and Bioinformatics Analysis

DNA extraction and amplification were carried out under physically separated and specifically dedicated laminar flow hoods to minimise contamination risk. Extractions were done using a QIAGEN DNeasy Blood & Tissue kit (Qiagen) following the manufacturer's instructions. The quality and quantity of purified amplicon were checked using the 2200 TapeStation (Agilent Technologies, Santa Clara, USA). We amplified the V4 region of the prokaryotic 16S rRNA gene (254 bp; Caporaso et al. 2011) and the V7 region of the eukaryotic 18S rRNA gene (123 bp; Gast, Dennett, and Caron 2004) using well‐established primer pairs. We selected these general primers as they amplify across all microbial eukaryote diversity. We are aware that this sequence length has limited resolution at the species level for fungi, protists and other algal groups. Still, it is nonetheless well‐suited to explore genus‐level diversity (De Vargas et al. 2015). Besides, the 18S allows more reliable quantification of fungi than the ITS region (Lepère et al. 2019). DNA amplification was carried out in single reactions, as recommended by Marotz et al. (2019), in two sequential PCRs using the Q5 High‐Fidelity polymerase 2× Master Mix (New England Biolabs). After each PCR, the amplified products were purified with HighPrep PCR beads (Ampure XP). Primers for the second PCR included unique sequencing barcodes as well as Illumina adapters. Following purification, DNA concentrations were measured, and amplification specificity was checked for all samples using gel electrophoresis. Three negative controls of sterile H_2_O were included during both extraction and amplification that in no case yielded detectable DNA concentrations (based on gel electrophoresis and measured DNA concentrations). The purified PCR products were pooled in equimolar concentrations and sequenced on the Illumina HiSeq for 16S gene at Centro Nacional de Análisis Genómico (CNAG‐CRG, Barcelona, Spain) and MiSeq platforms for the 18S gene at Macrogen (Macrogen Inc. Seoul, Republic of Korea).

The sequencing platform performed demultiplexing and provided a fastq file for each of the 206 libraries (103 sample libraries for water and 103 for biofilm samples). A first quality filtering step excluded DNA reads below 330 and 150 bp read length (for prokaryotes and eukaryotes, respectively), with a Phred quality score below 23 over a moving window of 25 bp, with more than one mismatch in the primer sequence and homopolymer over 8 bp, or with ambiguous base. All the fastq files were then treated together following the bioinformatics process described in Vasselon et al. (2017) using the Mothur software (Schloss et al. 2009). Reads that were not fully aligned with the SILVA (v132) database were removed. The 206 resulting files were analysed together. Chimera removal was done using the Uchime algorithm (Edgar et al. 2011). Taxonomic classifications of amplicon sequence variants (ASV) were performed with a similarity cut‐off of 97% (for prokaryotes) and 90% (for eukaryotes) against the SILVA (v132) database using the UCLUST algorithm. Singleton sequences were removed to reduce potential inflation of diversity due to sequencing errors. DNA reads assigned to ‘Eukaryota unclassified’ or ‘unclassified’ without information in any taxonomic rank were also removed for further analysis (22% of reads). Samples with several reads below 10.000 were discarded from the analysis (Aylagas et al. 2021; Grosser et al. 2019). We explored biodiversity patterns from ASV because ASV outperforms OTU method for estimating richness in prokaryotes and eukaryotes (e.g., fungi; Joos et al. 2020).

### 2.4 Environmental Variables

We analysed environmental variables in three groups: (i) climatic, hydrological and geological variables related to the catchment regional characteristics, (ii) topographical variables related to the river network and (iii) LULC variables. All environmental variables were integrated into a synthetic river network for each of the identified river reaches by running NetMap's virtual watershed algorithms (Barquín et al. 2015). Netmap is an integrated suite of numerical models and analysis tools that develops geospatial solutions for a wide range of applications for river ecosystems, such as hazard mitigation, watershed restoration or conservation (Barquín et al. 2015). The river networks were delineated using flow directions inferred from the available DEMs (Figure 1), using the algorithm described in Clarke, Burnett, and Miller (2008). Each river network was divided into reaches of length between 100 and 500 m and always divided into confluences, as these can produce important morphological changes in the channel and flood zones (Benda et al. 2004). All environmental variables were derived from each of the reaches of each river network.

For the regional variables, we measured climate, geology and *X*. Climatic variables for all case studies were derived from different data sources (Fonseca et al. 2022) and included mean annual precipitation (MN_rr) and mean maximum, minimum, average temperature and mean temperature during summer (MN_tx, Mn_tn and MN_tg, TMESU, respectively). For the Portuguese case study, a previously developed high‐resolution climate dataset, PT.HRES was used (Fonseca et al. 2022). For the remaining case studies, climatic data were retrieved from the E‐OBS v20e database at a ~10 km spatial resolution (Cornes et al. 2018) and resampled to produce the targeted, refined spatial resolution (~1 km spatial resolution).

Topographic variables were derived from National Digital Elevation models (DEM) for each country, with a 5 m spatial resolution. Global ASTER GDEM V2 (90 m) was used when no other information was available (see Table S1). See Table S2 to find data sources per country. From DEM, we derived the following topographic variables by using Netmap tools: the average elevation of the river reach (ELEV_M), the distance from the basin outlet (OUT_DIST), stream order (STRM_ORDER) and catchment area (AREASQKM). Lithological variables were derived from digital cartography based on regional national maps provided by local stakeholders and SOILGRIDS database (de Sousa et al. 2020) as reference information and included four rock types: igneous (MN_IG), limestone (M_LM), sedimentary (MN_SD) and schists (MN_SQ) (see Table S1). Regarding hydrology, we considered two main variables: mean flow during summer in each reach (FLOW) and number of high flow events per year using an upper threshold of three times the median flow over all years (FRE3; Table S1). They were derived from the Spatial Processes in Hydrology (SPHY) model, a hydrological modelling tool developed by Terink et al. (2015), and applied to the different case studies using the national gauge stations network and local river monitoring systems in each country. The mean generic erosion potential (MN_gepdelm and BF_gepdelm) was determined to identify potential sediment production, transport and deposition areas (Hernández‐Romero et al. 2022). NetMap's shallow landslide potential index can be used to consider the potential for processes that range from shallow landslide, and debris flow to gullying and sheet washing in convergent areas on hillsides.

LULC parameters were derived from regional LULC maps obtained from remote sensing and local occurrence data, using CORINE Land Cover 2018 map (CLC) from the Copernicus Land Monitoring Service (CLMS) as a baseline to produce coherent information at the European scale (Álvarez‐Martínez, Silió‐Calzada, and Barquín 2018). By running NetMap tools over LULC patterns, we have included four land cover categories in the assessments: mean broadleaf forest area (MN_blf) and (BF_blf); mean area of planted coniferous forest, *Eucalyptus* spp. or other plantations (sylviculture areas and labelled as MN_syl and BF_syl); mean agricultural area (MN_agr and BF_agr) and mean urban area (MN_uhd and BF_uhd). LULC variables were expressed as the percentage area that is covered by each class at two spatial scales: (a) MN—the drainage basin, catchment scale, and (b) BF—reach scale measured as 40 m buffer polygons to the reach that represented each river section (Pérez‐Silos, Álvarez‐Martínez, and Barquín 2019). Averages of environmental variables per catchment are available in Table S3.

### 2.5 Data Analysis

Water and biofilm taxa inventories were merged after bioinformatic processing and prior to any statistical analyses into a single sample for each surveyed river reach. All analyses were evenly performed for prokaryotes, fungi, heterotrophic protists and algae (autotrophs belonging to the Protists and Plantae kingdom).

Before the analysis leading to testing the hypothesis, we performed species accumulation curves and compositional treemaps, checked estimated diversity across catchments and tested if differences in RMC diversity existed between catchments to have an overview of the prokaryote and eukaryote dataset in the Atlantic catchments. To test differences in microbial diversity between catchments, we used one‐way ANOVAs and Tukey post hoc tests once we checked that assumptions of normality and homogeneity of variances were met. Tests were done for each taxonomic group. Because ASV richness in next‐generation sequencing is highly sensitive to differences in the number of sequences obtained across samples, we used rarefaction analysis to the smallest sequencing depth within each group to obtain a robust comparison of relative richness between samples using the function ‘rarefy’ in vegan (Oksanen et al. 2013). This function determines the species detected in random subsamples of identical sequence counts. Rarefaction curves were performed with the function ggrare from the ranacapa package (Kandlikar et al. 2018). A non‐parametric richness index (Abundance‐based Coverage Estimator; ACE) was calculated to estimate the true richness of all groups at the catchment scale. ACE is the most appropriate richness index in this study because it estimates the proportion of all individuals in rare species that are not singletons (Magurran and McGill 2010). Correlation matrices of richness and environmental variables were created to test the effect of large and catchment scale variables on RMC diversity (Hypotheses i and ii), using rcorr R function and corrplot package. A full multiple regression model has been done with all environmental variables and variable selection was performed using a stepwise model based on AIC criteria. Finally, variation partitioning of environmental variables and richness of RMC was done using the function varpart from the vegan package.

For compositional analysis, ASV matrix was Hellinger transformed. ASV with very low abundances were discarded for compositional analyses while retaining 98% of cumulative read abundance (ASV with abundances > 0.005 for prokaryotes and > 0.001 for eukaryotes). To test if distinct RMC can be detected among catchments (Hypothesis i) we used a permutational multivariate analysis of variance (PERMANOVA) with the function ‘adonis’ based on Bray Curtis distance using vegan package (Oksanen et al. 2013), and using catchment type as the explanatory variable. To further perform multilevel pairwise comparisons, the function ‘pairwise.adonis’ (Martinez Arbizu 2020) was applied. Correlation of compositional matrices was done using Mantel test based on Bray Curtis distance as applied in the vegan package.

The effect of environmental variables on microbial composition was tested with multivariate analysis (leading to test Hypotheses i and ii). Environmental variables were log_10_ converted. Some variables were strongly correlated with others (*R* ^2^ > 0.7) and were removed from the redundancy analysis (RDA). These variables were: river width (WIDTH_m) correlated with catchment area (AREA_SQKM), mean minimum temperature (Mn_tn), mean maximum temperature (MN_tx) and mean temperature during summer (TMESU), correlated with mean average temperature (MN_tg), mean flow during summer (FLOW) correlated with catchment area (AREA_SQKM). Environmental variables were divided into three groups: (1) variables related to the regional identity of the catchment (climate, geology and hydrological variables); (2) topographical variables (related to the downstream gradient or network position); and (3) LULC variables.

For testing the hypothesis related to disentangling which group of environmental variables was the most important in shaping ASV composition, we performed RDA to explore environment‐community relationships for both prokaryotes and eukaryotes at the highest level of taxonomic resolution possible (genus or lower). Based on these groups, a forward selection of environmental variables followed by a variation partitioning analysis was performed using CANOCO software (ter Braak and Šmilauer 2012) to calculate each group of variables' specific contribution per taxonomic group and their interaction. With the above‐mentioned selection procedures, the significant environmental variables were merged to perform the final RDAs. Finally, Pearson correlations between all predictors and the most abundant taxa per group (taxa with relative abundances > 0.3% for prokaryotes and > 0.2% for eukaryotes) were performed with the function cor_heatmap.

All data analyses except ordination analysis were performed in R (R Core Team 2021). In addition, R packages ggplot2 (Wickham 2011), phyloseq (McMurdie and Holmes 2013), lattice (Sarkar, Sarkar, and KernSmooth 2015), corrplot (Wei et al. 2017), microbiome (Gilmore et al. 2019) and ggrepel (Slowikowski et al. 2018) were used for transforming and manipulating ASV tables and visualising statistical results.

## 3 Results

### 3.1 High‐Throughput Sequences Processing and Taxonomic Assignment

**FIGURE 2:**
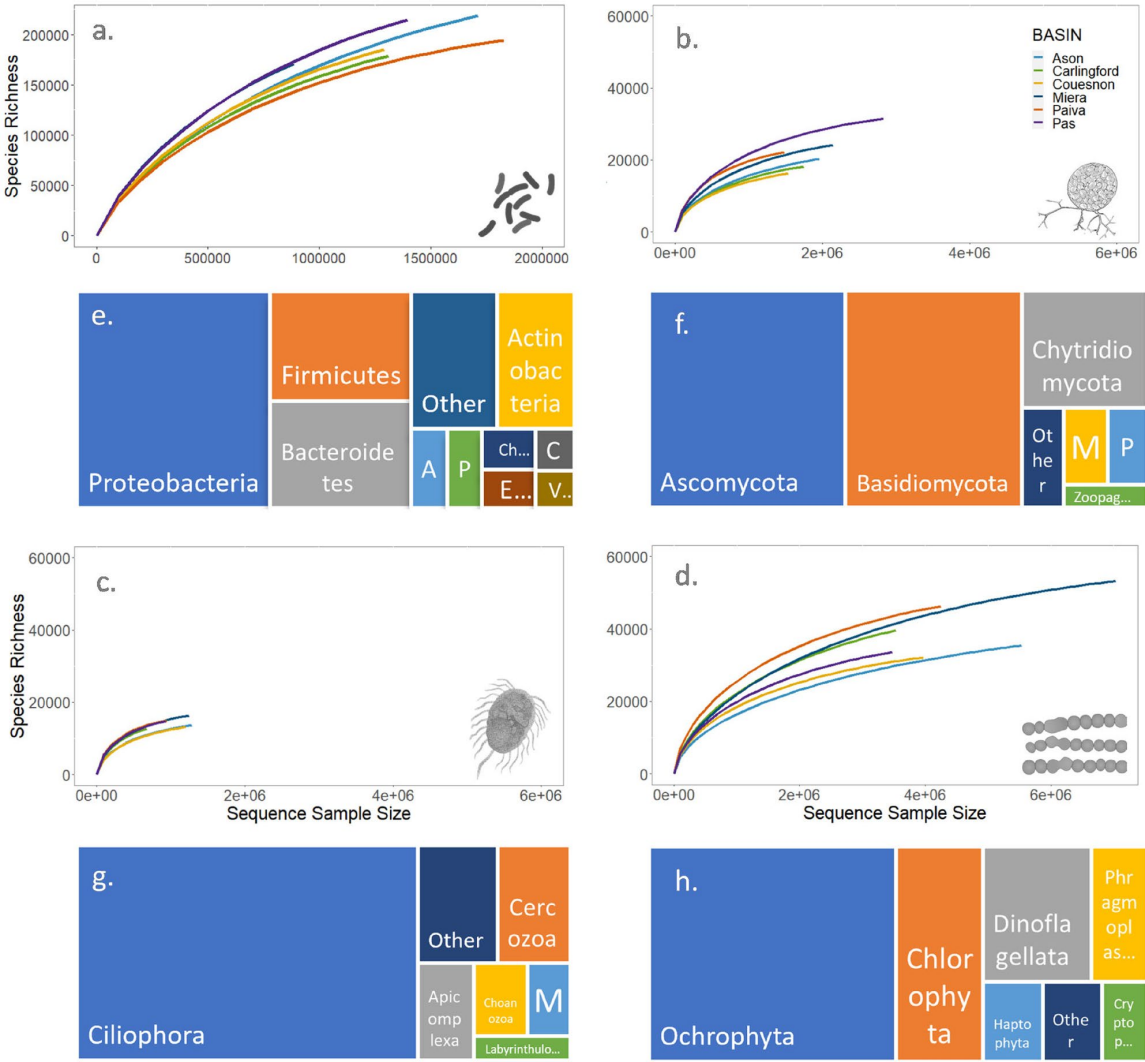
Overview of the prokaryote (V4 16S rDNA) and eukaryote (V7 18S rDNA) datasets in the Atlantic catchments. Sequence size rarefaction curve for each of the analysed groups (a–d) and treemaps representing the diversity (size of rectangles) of the most abundant phyla per taxonomical group ((e) prokaryotes, (f) fungi, (g) heterotrophic protists and (h) algae). Panel e—A: Acidobacteria; C: Cyanobacteria; Ch: Chloroflexi; E: Euryarchaeota; P: Planctomycetes; V: Verrucomicrobia. Panel f—M: Mucoromycota; P: Peronosporomycetes; Zoopag: Zoopagomycota. Panel g—Labyrinthulo: Labyrinthulomycetes; M: Mast‐3. Panel h—Cryptop: Cryptophyta.

We obtained 8.679.718 reads grouped in 24.769 and 193.674 ASV averaged per sample and catchment, respectively, for prokaryotes. Average number of reads per sample was 86.797,187. Rarefaction analysis indicated that ASVs did not approach saturation at the catchment level, although a reduction on slopes was observed at the end of each rarefaction curve in prokaryotes and eukaryotes (Figure 2). ACE estimated the true prokaryote richness to 241.000–311.000 ASVs, suggesting that our survey retrieved ∼60%–80% of prokaryote diversity at the catchment level over the surveyed Atlantic bioregion (Table 1). ASVs were affiliated to 2216 taxa, from which 1401 were genera, and the rest were classified to lower taxonomic levels. The prokaryote phyla with the higher diversity were Proteobacteria (representing 39%), Firmicutes (14%), Bacteroidetes (14%) and Actinobacteria (10%; Figure 2e). Regarding eukaryotes, protists accounted, on average more than 60% of total eukaryotic ribosomal diversity when considering all samples, followed by Fungi (ca. 30%) and Plantae kingdom (5%). Within the Fungi kingdom, we obtained 11.712.745 reads grouped in 3.218 ASVs and 20.122 ASVs averaged per sample and catchment, respectively. Average number of reads per sample was 118.310. ACE richness estimator extrapolated the true fungal richness to 21.000–36.500 ASVs in the catchments analysed, suggesting that our survey retrieved ∼75%–86% of fungal richness at the catchment scale (Table 1). The ASVs were affiliated to 287 taxa. The fungi phyla with the higher diversity in terms of the number of genera were Ascomycota (representing 40%), Basidiomycota (35%), Chytridiomycota (13.5%) and Mucoromycota (3%; Figure 2f). The heterotrophic protists yielded 6.276.745 reads grouped in 1.860 ASVs and 14.231 ASVs averaged per sample and catchment, respectively. Average number of reads per sample was 63.401. ACE richness estimator suggested a total heterotrophic protist richness in these catchments between 16.000 and 19.900 ASVs. Thus, our survey unveiled ~76%–86% of heterotrophic protist ribosomal diversity on average in the analysed Atlantic catchments (Table 1). The ASVs were affiliated to 136 taxa. The phyla with the highest richness in terms of the number of genera were Ciliophora (69%), Cercozoa (8%), Apicomplexa (5%) and Choanozoa (4%; Figure 2g). Finally, within the algae group (phototroph protists and kingdom Plantae), we obtained 27.802.008 reads grouped in 5.230 ASVs and 40.000 ASVs averaged per sample and catchment, respectively. Average number of reads per sample was 280.800. ACE richness estimator extrapolated the true algae richness to 40.500–65.500 ASVs in the catchments analysed, suggesting that our survey retrieved ∼72%–80% of algae richness at the catchment scale (Table 1). The ASVs were affiliated with 270 taxa. The phyla with the higher diversity genera were Ochrophyta (50%), Clorophyta (17%), Dinoflagellata (14%) and Phragmoplastophyta (7%; Figure 2h).

**TABLE 1:** ASV richness for the six catchments, showing rarefied richness, observed richness, output of ACE richness estimators and percentage of richness retrieved per catchment in each of the microbial groups (prokaryotes, fungi, heterotrophic protists and algae).

		Rarefied richness	Observed richness	ACE	Richness retrieved (%)
Prokaryotes	Ason	158,015.00	158,015	311,055 ± 325	70
	Miera	171,053.00	171,053	288,492 ± 347	59
	Pas	173,272.10	214,540	304,491 ± 323	70
	Carlingford	149,144.10	178,471	253,854 ± 295	70
	Paiva	142,982.70	194,422	241,879 ± 247	80
	Couesnon	155,346.40	185,082	267,268 ± 306	69
Fungi	Ason	18,368.82	20,318	27,231 ± 80	75
	Miera	21,325.91	24,127	30,333 ± 88	80
	Pas	25,556.36	31,404	36,426 ± 89	86
	Carlingford	17,227.20	18,212	23,701 ± 81	77
	Paiva	22,168.94	22,168	26,406 ± 78	84
	Couesnon	16,089.02	16,267	21,028 ± 75	77
Protist	Ason	10,920.56	13,625	17,015 ± 65	80
	Miera	13,366.57	16,302	19,794 ± 69	82
	Pas	13,363.82	14,747	18,095 ± 66	81
	Carlingford	12,601.00	12,601	16,607 ± 66	76
	Paiva	13,606.94	14,944	17,404 ± 61	86
	Couesnon	10,792.06	13,168	16,262 ± 62	81
Algae	Ason	29,651.66	35,423	48,853 ± 122	72
	Miera	41,217.52	53,526	65,487 ± 127	81
	Pas	33,608.00	33,608	43,473 ± 108	77
	Carlingford	39,398.07	39,648	55,020 ± 129	72
	Paiva	43,523.30	46,275	57,829 ± 122	80
	Couesnon	30,918.90	32,184	40,670 ± 103	79

### 3.2 RMC Diversity and Composition Across Catchments

**FIGURE 3:**
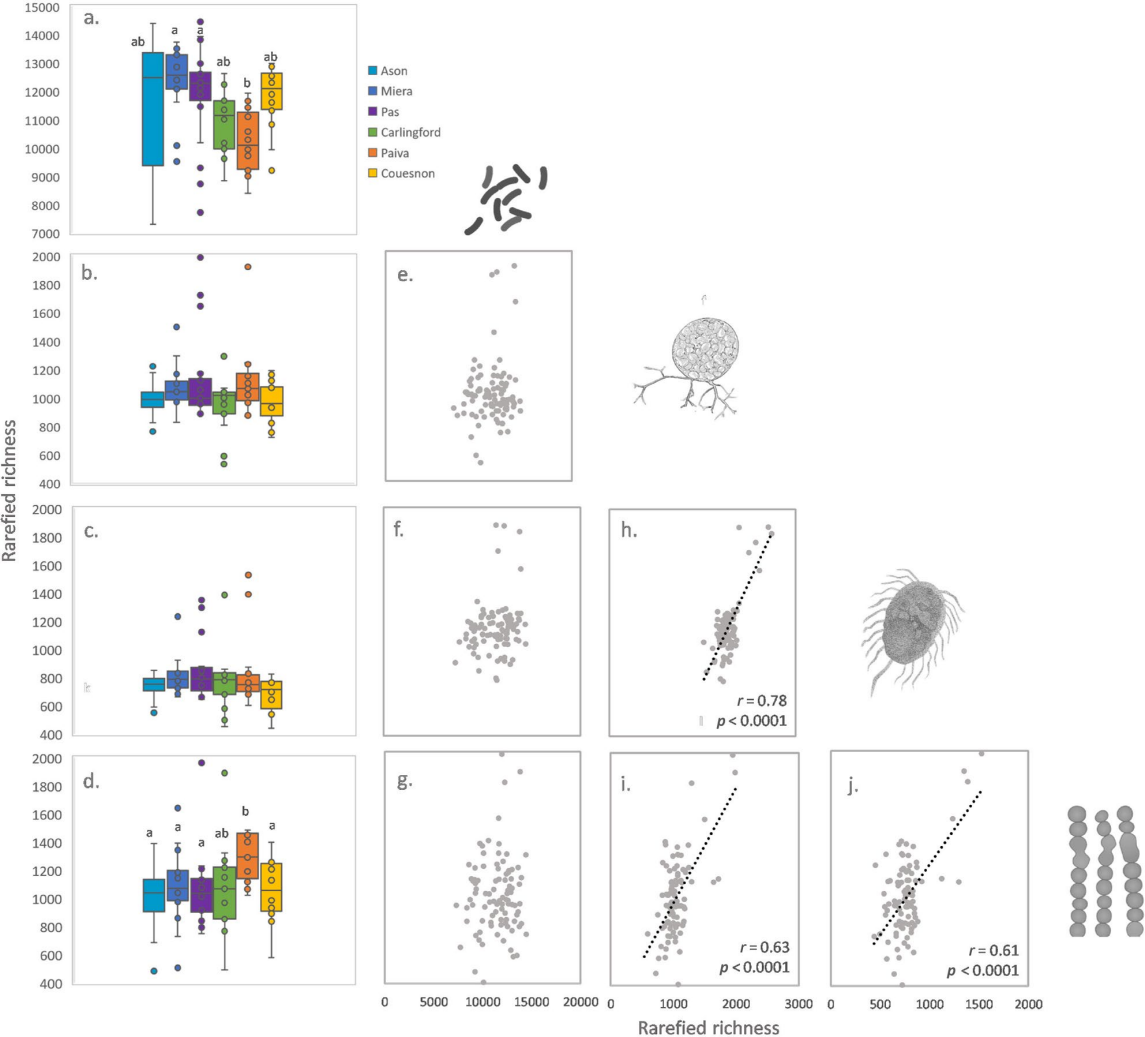
(a–d) Variation in rarefied richness (expressed as number of ASVs) across catchments. Letters denote statistically homogeneous groups based on ANOVA. (e–j) Rarefied richness correlations (Pearson) between RMC groups.

When comparing the rarefied richness of all the groups between catchments, we found significant differences within prokaryotes (ANOVA *F* = 4.17 *p* = 0.002) and algae (ANOVA *F* = 3.86 *p* = 0.003; Figure 3) and not within fungi (ANOVA *F* = 2.23 *p* = 0.06) or protist (ANOVA *F* = 1.38 *p* = 0.23). The Tukey post hoc test revealed that the significant results were due specifically to lower bacterial richness in Paiva in comparison to Miera and Pas for prokaryotes, and due to larger algal diversity in Paiva in relation to algal diversity of Ason, Pas, Miera and Couesnon (Figure 3 and Table S4). We found consistent patterns of diversity within eukaryotes across Atlantic catchments, denoted by significant Pearson correlations (Figure 3h–j). No relationship were found between the diversity of prokaryotes and any of the eukaryotic groups (Figure 3e–g).

**FIGURE 4:**
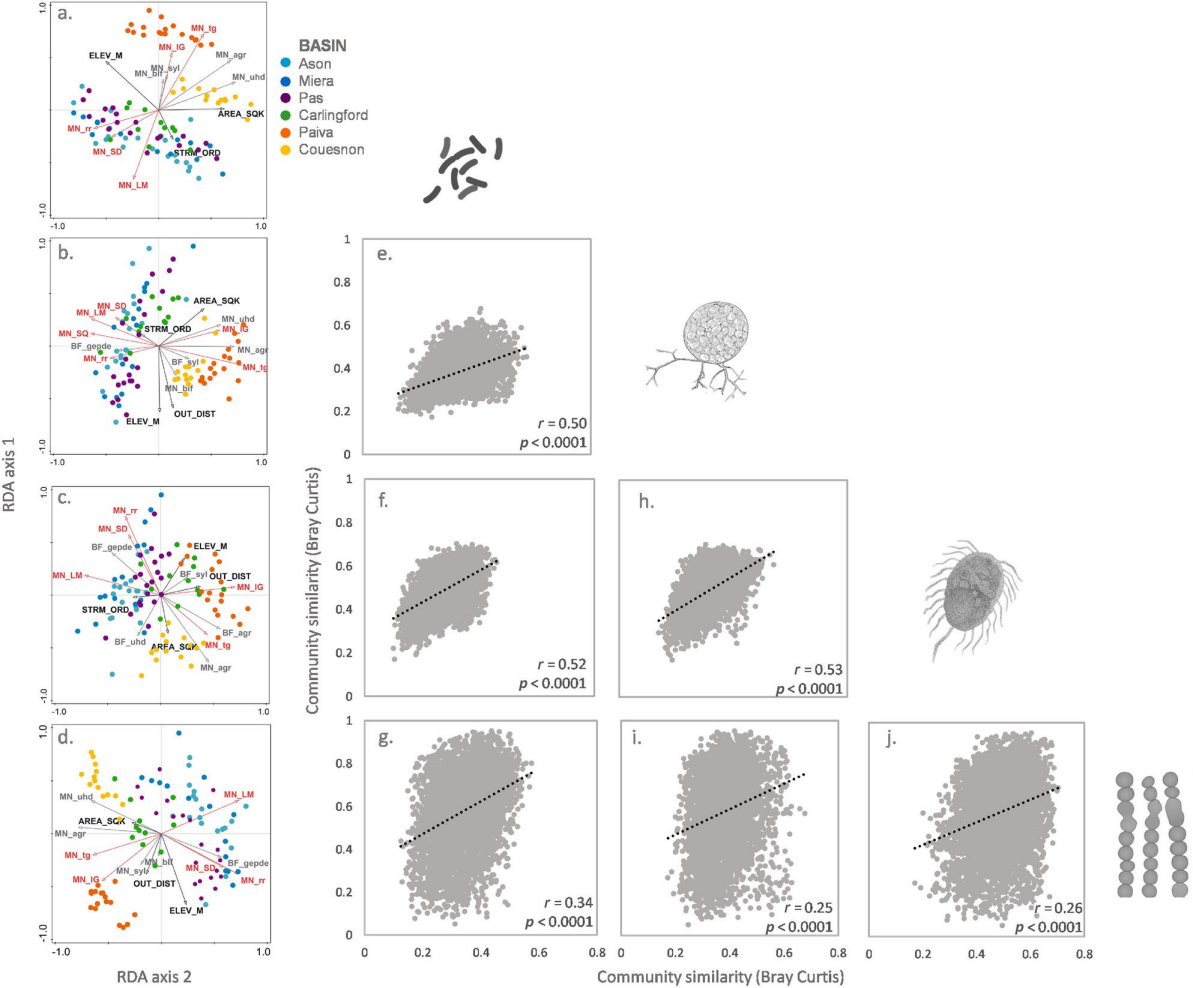
(a–d) Redundancy analysis (RDA) between microbial communities (prokaryotes; fungi; heterotrophic protists and algae) at highest level of taxonomic resolution possible and environmental variables. For each of the analysed communities, environmental variables were selected via redundancy analysis followed by forward selection separately per group of variables: (1) climatic‐geological (red), (2) topographical (black) and (3) LULC (grey). Then, the selected variables were used for final redundancy analysis. See Table S1 for environmental variable codes. (e–j) Correlations of community composition (Mantel tests based on Bray Curtis similarity).

The analysis of community similarity (based on the PERMANOVA test) revealed statistically significant differences among the six catchments for all the considered organism/taxonomic groups: prokaryotes (*F* = 7.67; *p* = 0.001), fungi (*F* = 6.34; *p* = 0.001), protist (*F* = 4.01; *p* = 0.001) and algae (*F* = 7.05; *p* = 0.001; Table S5). Sites from Paiva were the most dissimilar from all studied catchments. The pairwise adonis test revealed significant differences for the communities of fungi, protists and algae across the six catchments, although there were no significant differences among the three Spanish catchments and between Pas and Carlingford Lough (Table S5). We found significant correlations between all group responses regarding community composition (mantel test based on Bray Curtis similarity, Figure 4e–j).

### 3.3 Environmental Drivers of RMC Diversity

**FIGURE 5:**
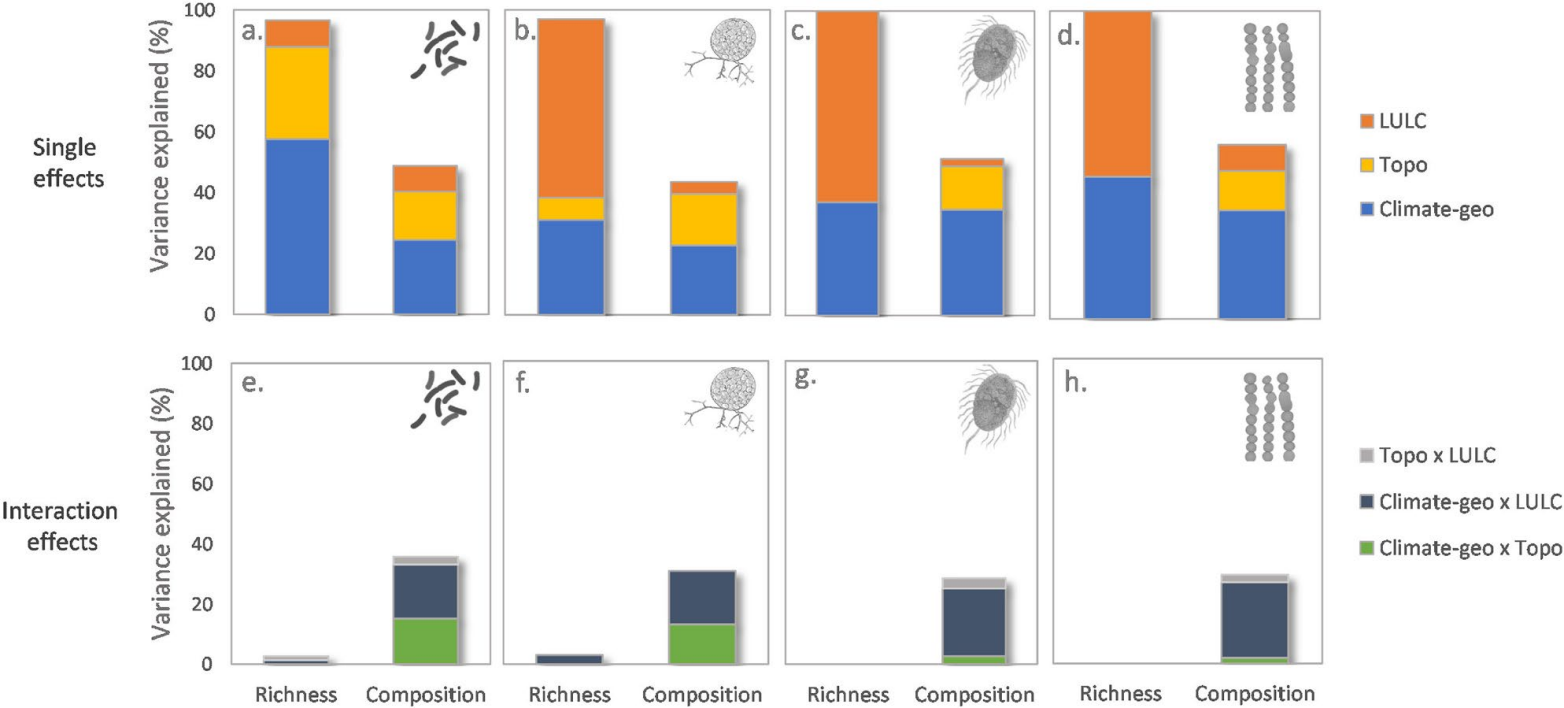
(a–d) Variation partitioning results denoting the percentage of unique explained variation per group (single effects) of environmental variables (climatic‐geological, topographical and LULC) for RMC richness and composition. (e–h) Percentage of shared variation (interaction effects) of the three groups of variables for RMC richness and composition.

Richness models selected by AIC criteria showed that geological variables were important in explaining richness for all the groups but also highlighted some differences. For example, basin area (AREA_SQKM) was the most important for prokaryotes while LULC was more relevant for explaining richness in eukaryotes (Table 2; Table S9). Variation partitioning for richness patterns showed that the most relevant fractions shaping RMC diversity were the individual fractions as the interactive effects were very low. The drivers for diversity in prokaryotes were, in order of importance, geological (23% explained individually), topographical (12% explained individually) and LULC (3.6% explained individually; Table S6). Eukaryote diversity, in contrast, were driven mostly by LULC variables (with 13.8%, 12.8% and 14.7% of explained variance by LULC fraction individually for fungi, protists and algae; Figure 5 and Table S6) and by climatic‐geological variables in the second place (7.4%; 7.5% and 12.6% for fungi, protist and algae, respectively). Topographical variables were only significant for prokaryote and fungal diversity (AREASKQM for both, and elevation was only significant for prokaryote diversity; Table S9).

**TABLE 2:** Richness model parameters per group (prokaryotes, fungi, heterotrophic protists and algae) on best model selected according to AIC criteria. *F*‐statistic, adjusted *R* ^2^ and *p* values are also shown.

Group	Model parameters for richness	*F*	Adj *R* ^2^	*p*
Prokaryotes	AREASQKM, FRE3, MN_uhd, MN_IG, MN_SD, MN_SQ, MN_syl, MN_tg, ELEV_M	7.216	0.37	< 0.0001
Fungi	AREASQKM, MN_blf, BF_blf, BF_agr, BF_gepdelm, MN_IG, MN_LM, MN_SD, MN_SQ	3.261	0.175	0.0018
Protists	BF_blf, MN_uhd, BF_gepdelm, MN_SQ, MN_SD, MN_rr	4.316	0.172	0.0007
Algae	BF_blf, MN_uhd, BF_gepdelm, MN_SQ, MN_syl	6.41	0.22	< 0.0001

### 3.4 Environmental Drivers of RMC Community Composition

Regarding community composition, all three groups of environmental variables (climatic and hydrogeological, topographical and LULC) were significant. The total explained variation of the RDA analysis was highest for prokaryotes (35%) followed by algae (28.3%), fungi (24.5%) and protists (18%; Figure 4; Table S7). The analysis of variation partitioning revealed that the highest percentage of unique explained variation was for climatic‐geological variables (24.6% for prokaryotes; 22.7% for fungi; 34.8% for protists and 35.4% for algae), being average temperature and precipitation most important for bacteria and fungi, while the percentage of limestone was the most important for protists and algae (Figure 5 and Table S7). The next group in order of importance was the group of topographical variables (outlet distance, catchment area, stream order and elevation; 16% of the unique explained variation for prokaryotes, 17% for fungi, 14% for protists and 13% for algae, Figure 5 and Table S7). The last environmental variable group was related to LULC, and the percentage of unique explained variation was 8.4% for prokaryotes, 3.8% for fungi 2.5% for protists and 8.4% for algae (Figure 5 and Table S7). The variable with the highest significant contribution for all the biological groups was the percentage of agricultural area in the catchment (MN_agr), although the mean generic erosion potential (MN_gepdelm) and the mean percentage of urban area (MN_uhd) in the catchment was also significant for most biological groups (all of them except bacteria for mean generic erosion potential; Table S8). To a lesser extent, the mean percentage of sylviculture was significant for all the groups, and broadleaf forest areas were significant for all except protists (Table S8). The variation partitioning analysis also revealed that an important part of the variance explained was due to the interaction effects. This was especially relevant for climatic‐geological and LULC for all the groups (between 17.5% and 25% of explained variation, Table S7) and also between climatic‐geological and topographical for bacteria and fungi (between 13% and 15%, Table S7).

**FIGURE 6:**
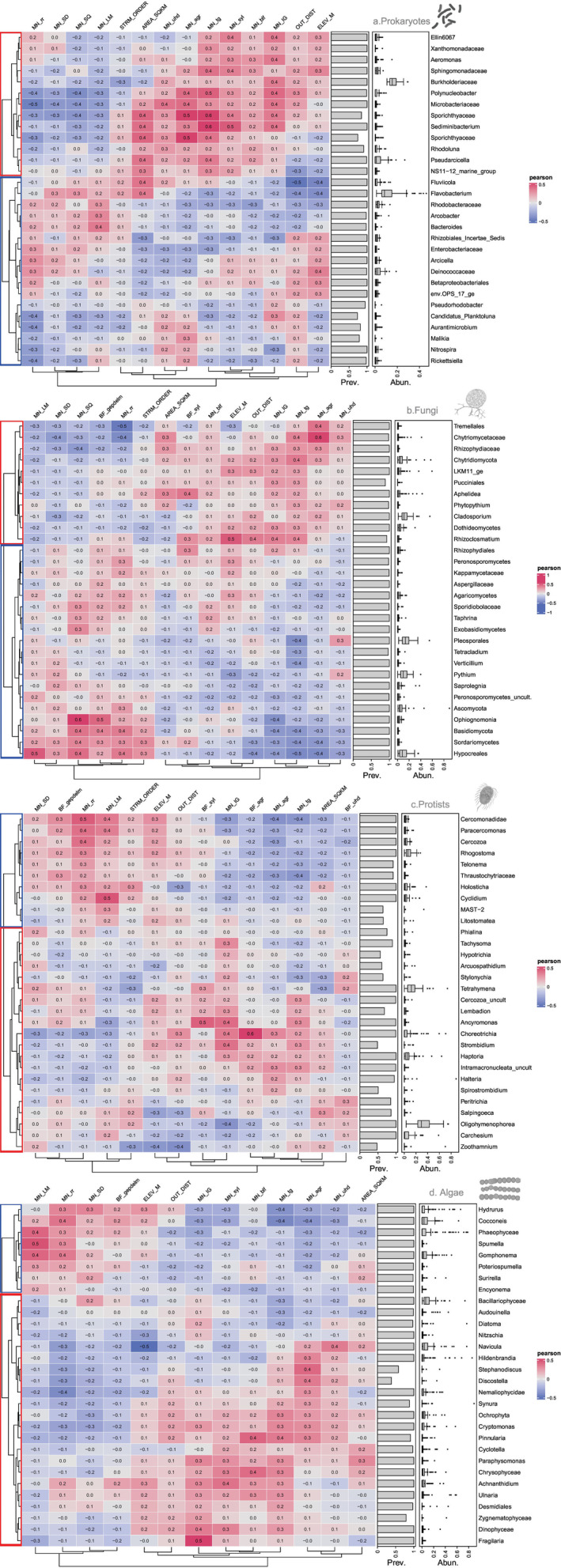
Correlation heatmaps showing the Pearson correlation between the significant environmental variables based on RDAs and the top 30 most abundant taxa per taxonomical group: (a) prokaryotes, (b) fungi, (c) heterotrophic protist and (d) algae. Column dendrograms denotes correlation between environmental variables and row dendrograms denote taxa correlations. Taxa prevalence (Prev.) and relative abundance (Abun.) is also shown per taxonomical group. The first division of row dendrograms split the taxa in two groups: red rectangles denote communities more abundant in warmer catchments (Couesnon and Paiva) while blue rectangles denote communities more abundant in catchments with lower average temperature and regular year rainfall (Spanish catchments and Carlingford Lough). See Table S1 for environmental variable codes.

Pearson correlations of environmental variables and communities highlighted two main groups for variables (column dendrograms) and taxa (row dendrograms; Figure 6a–d), separating warmer catchments RMC (Paiva and Couesnon—red rectangles; Figure 6) from the RMC more prevalent in the Spanish and Carlingford Lough catchments with lower average temperatures and regular rainfall (blue rectangles; Figure 6). For prokaryotes, we have detected consistent responses of taxa and environmental variables at the order level. For example, we have found taxa from orders Sphingomonadales, Aeromonadales and Betaproteobacteriales (Burkholderiaceae) positively related to increases on temperature. Taxa from Chitinophagales (*Sediminibacterium* sp.), Micrococcales, Frankiales (Sporichthyaceae) or Cytophagales (*Pseudarcicella* sp.) were correlated to both temperature and agricultural areas. Some taxa were more abundant under higher elevations for example Betaproteobacteriales (Ellin6067) or Sphingomonadales. In relation to fungi, some generalisations were possible at the phylum level. For example, we found the phylum LKM15 positively related to temperature, while Chytridiomycota and Blastocladiomycota were positively related to both temperature and agricultural area in the catchment. It was also noticeable that these taxa were found predominately in lower reaches. From other phyla, taxa from classes Dothideomycetes, Peronosporomycetes (*Phytopythium* sp.) or Pucciniomycetes (order Pucciniales) were also significantly related to temperature and, at the same time, more abundant at stream reaches with higher altitude. The protistan taxa favoured by higher temperatures were *Lembadion* sp. (Ciliophora) and Ancyromonadida class. In addition, affected positively by both, temperature and agricultural areas were mostly ciliates (Haptoria, Choreotrichia and *Strombidium* sp.). In general, heterotrophic protists were more abundant at the lower part of the river networks, especially ciliates (*Peritrichia* sp., *Salpingoeca* sp., *Carchesium* sp.).

The algae in the warmer catchments (Paiva and Coueson) were split into two groups: (1) related to higher temperatures and larger agricultural areas, mainly from Cryptophyceae class (*Synura* sp. or *Cryptomonas* sp.), and (2) significantly correlated to temperature but also igneous rocks (dominant at the Paiva catchment) for example Dinoflagellata and Zygnematophyceae classes and some diatoms that increased with elevation (*Achnanthidium* sp., *Ulnaria* sp.). On the other hand, taxa dominant in the lower reaches were centric diatoms (*Cyclotella* sp.) or taxa from t‑he Chrysophyceae class.

In the opposite group (Spanish catchments and Carlingford Lough marked with blue rectangles in Figure 6), we found taxa positively related to the most humid conditions and overall lower average temperatures. Prokaryotes from orders Rhizobiales, Enterobacteriales and Deinococcales were positively related with precipitation but also to elevation (Figure 6a), while Bacteroidales and Flavobacteriales were more prominent in lower reaches in areas with high average precipitation (Figure 6a). In relation to Fungi, the majority of taxa from phyla Ascomycota and Basidiomycota and all of taxa from Zoopagomycota were also positively related to precipitation, being some of them positively correlated to elevation (Peronosporomycetes, Agaricomycetes; Figure 6b). Protist taxa related to precipitation and limestone rocks were mainly included in the Cercozoa phylum (Figure 6c), being more prevalent in higher altitudes. Other protist phyla (Ciliophora, Labyrinthulomycetes or MAST‐2) were more prevalent in the lower parts of the reiver network (Figure 6c). Regarding algal groups, the ones positively related to precipitation and limestone rocks belong mostly to Chrysophyceae (*Hydrurus* sp., *Spumella* sp.), Phaeophyceae (Ectocarpales), Diatomea and Florideophyceae (red algae such as *Audouinella* sp.). Positively correlated to elevation stood out *Hydrurus* sp. and *Cocconeis* sp., while in lower reaches the abundance of *Poteriospumella* sp. or the diatom *Surirella* sp. (Figure 6d) increased markedly.

## 4 Discussion

The extent of the eDNA ALICE project dataset allows an unprecedented examination of the structure of RMC across biogeographical gradients over Atlantic landscapes. Here, we found evidence of divergent drivers for RMC diversity and composition. Environmental correlates of species richness were not common across taxa and were predonminantly related to topographical and geological variables for bacterial diversity and LULC for eukaryote diversity. This suggests that large‐scale factors are not the most important drivers of eukaryote diversity but factors such as land cover (forests) and land use (extension of agricultural or urban areas). Species composition, in contrast, was strongly correlated across taxa, with a number of the same environmental drivers being significant for most microbial communities. Spatial scale structured the observed compositional patterns of RMC communities, denoting the importance of environmental filters related to climatic and geological characteristics shaping RMC at European and regional scale, while the most important variables at the catchment scale were river network position (i.e., downstream gradient) and LULC.

The lack of correlation between prokaryote and eukaryote diversity has been found in other studies of freshwater organisms, as well as a high correlation between the richness of fungi and protist (Wood et al. 2017). Recent studies have revealed that a large fraction of protists also feed differentially on fungi (Geisen 2016) and that protist diversity, and community composition explained more variation in the beta diversity of certain saprotrophic fungi than abiotic factors (Huang et al. 2021). The high correlation between fungal and protistan richness thus, can be attributed to the modulating role that protists might have on fungal communities.

Species composition, in contrast, was strongly correlated between RMC groups. The observed co‐occurring patterns of all the groups analysed with environmental variables have been related to a Clementsian view of communities responding as coherent units (Wood et al. 2017). The correlation in community composition across all these disparate taxa implies an interconnected network of species that responds to the same environmental conditions according to the spatial scale and influence of each other's presence and abundance due to trophic interactions, mutualistic and competitive relationships.

### 4.1 16S and 18S rRNA Gene Sequences and Taxonomic Coverage

Our dataset, covered around ~9 and ~45 million reads and ca. 200.000 ASVs and 75.000 ASVs for prokaryotes and eukaryotes per catchment. As with many other biodiversity datasets built with metabarcoding techniques over large scales (Debroas et al. 2017; Malviya et al. 2016), the rarefaction analysis of prokaryotes and eukaryotes indicated that communities did not reach saturation. However, our extensive sampling effort (in terms of spatial extent and sequencing depth) gathered the majority (ca. 75%) of the expected diversity of prokaryotic and eukaryotic ribosomal diversity. Microbial diversity in terms of ASVs was higher in the prokaryote community than in the eukaryote community, as found in other studies on river biofilms (Sun et al. 2014; Guo et al. 2021). Regarding eukaryotes, the Atlantic rivers have shown a prevalence of protist rDNA biodiversity compared to classical multicellular eukaryotes, that is, animals, plants and fungi. Protists accounted for ca. 60% of total eukaryotic freshwater ribosomal diversity, which may indicate a lower proportional richness of protists in river ecosystems concerning marine environments (> 85%; De Vargas et al. 2015). Fungi, conversely, comprised ca. 30% of eukaryote richness, somewhat higher than the previously reported contribution of fungi to overall eukaryotic richness in freshwater environments (ca. of 17%; Lepère et al. 2019).

### 4.2 Drivers of RMC at the Regional Scale: Climate and Geology

Large scale geological variables turned out to be significant in explaining the diversity of RMC, especially for bacteria, while climatological variables did not impact significantly the diversity of RMC. In contrast to metazoans, a substantial portion of bacterial aquatic diversity within surface freshwaters may originate in soil environments (Crump, Amaral‐Zettler, and Kling 2012). In this regard, headwaters have been suggested to collect microorganisms from terrestrial sources (Crump, Amaral‐Zettler, and Kling 2012) which can contribute to community assembly of benthic bacterial biofilms (Besemer et al. 2013). Headwaters are intimately connected to the terrestrial environment (England and Rosemond 2004) and are characterised by a large ratio of benthic surface area to water volume, relative to larger fluvial ecosystems downstream. This might explain why geological variables such as igneous, sedimentary and schist explain a substantial part of the bacterial richness model.

While RMC diversity varied significantly only between some of the groups between catchments (algae and bacteria), the dissimilarity of community structure varied significantly between the Atlantic catchments for all organism groups considered. This low compositional dissimilarity for all groups suggests a convergence of communities driven by climatic characteristics, specifically lower average annual temperatures and regular rainfall throughout the year. This consistent driver for all organism groups highlights the importance of the environmental filter comprising large‐scale climatic factors in determining the regional species pool. In particular, climate was more determinant than geology for the composition of bacteria and fungi, while geological characteristics (% of limestone) were more relevant for the composition of protists and algae.

Temperature is one of the primary factors driving biological community composition worldwide (Zeglin 2015; Liu et al. 2019; Chen et al. 2018; Yang et al. 2019). Specifically, temperature was the second most important variable after metals for determining the composition of stream bacteria according to the review of Zeglin (2015) and one of the most important in the study of Liu et al. (2019) and Ruiz‐González et al. (2013). Similarly, the effect of temperature in other microorganisms such as fungi, protists or algae is also determinant in shaping the distribution of these communities at large scales (Zhang et al. 2018; Xiao et al. 2018). Geology, on the other hand, determined the potential range of proximate physicochemical variables such as nutrients, conductivity and pH, which are widely known to be key variables shaping the distribution of many microbes such as phagotrophic protists and other algae (e.g., diatoms and green algae; Potapova and Charles 2002). For example, limestone turned out to be one of the most important geological variables, may be due the relatively high export of dissolved ions in limestone dominated catchments.

#### 4.2.1 Potential Indicators of Temperature Changes

We highlighted in our study taxa whose presence and abundance were related to warmer temperatures in all taxonomic groups. For instance, temperature had a positive influence on the abundance of several bacterial taxa from the orders Chitinophagales (e.g., *Sediminibacterium* spp.) and Frankiales (family Sporichthyaceae). These taxonomical orders dominate streams during the warmest months (Engloner et al. 2023) and can survive under very high temperatures (Rincón‐Molina et al. 2019; Chaudhary and Kim 2016). The abundance of other bacterial taxa such as Betaproteobacteriales and some Bacteroidetes has been reported to increase with global warming (von Scheibner et al. 2014), which were also correlated with temperature in our study. We found that temperature has also a positive influence on fungi from phylum Ascomycota (*Bipolaris* spp. or *Acremonium* spp.), Aphelidiomycota or Chytridiomycota, heterotrophic protists from the order Litostomatea (Haptoria) and phototroph protists such as dinoflagellates. Fungi from phylum Chytridiomycota has been reported to be the most dominant group in streams with water temperature above 8.6°C (Seena et al. 2019). Dinoflagellates were especially favoured by higher temperatures while lower temperature generally favoured some species of diatoms (Xiao et al. 2018). A warming effect and a trend of increasing eutrophication might promote dinoflagellates over diatoms (Xiao et al. 2018). High water temperature (20°C–27°C) was also positively affecting the occurrence of *Cryptomonas* spp. (Phylum Cryptophyta), as it has been found that is favourable for its reproduction (Zhu, Bi, and Hu 2013). In addition, we found that temperature rise can be detrimental for some other taxa, for example, the brown algae *Hydrurus* sp., which is ecologically important as a food source for macroinvertebrates and does not tolerate temperatures above 10°C–12°C (Stanković and Leitner 2016).

### 4.3 Drivers of RMC at the Catchment Scale: Topographical Variables and LULC

Once the environmental filter of climatic and geological constraints has been passed, RMC responded primarily to topographical variables related to river network position (i.e., downstream gradient) and land use variables which in many cases are intimately related, demonstrating the occurrence of biogeographical patterns at the river network level. In relation to this, it is worth noting that drainage area upstream of the site was the best predictive spatial feature for bacterioplankton richness compared to other physical and chemical variables (Read et al. 2015; Besemer et al. 2013). In agreement with our study, numerous studies on rivers have found that upstream river reaches amassed high bacterial species richness, before richness decreased further downstream (Crump, Amaral‐Zettler, and Kling 2012; Savio et al. 2015; Niño‐García, Ruiz‐González, and Del Giorgio 2016). This pattern contrasts with the theoretical predictions of the RCC and other patterns reported from studies on invertebrate and fish assemblages (Besemer et al. 2013). Microbial communities from headwater reaches are heavily influenced by a large input of allochthonous organisms from soils and groundwater into the river network, as it was mentioned in the previous section. As the rivers progresses, such mass effects likely heeded to species sorting, a process requiring an increased water residence time to allow for the selection of species based on local environmental conditions (Crump, Amaral‐Zettler, and Kling 2012; Savio et al. 2015; Niño‐García, Ruiz‐González, and Del Giorgio 2016). Moreover, changes in microbial community composition from upstream to downstream sections were also significant and can be linked to the important downstream hydrological and biogeochemical changes along the river continuum but at the same time, to the intensity of disturbances due to human activities (e.g., changes in land use or vegetation; Leira and Sabater 2005; Henson et al. 2018).

In relation to eukaryotic community composition, the highest percentage of unique explained variation in relation to the longitudinal river gradient was found for fungal taxa, although their effect was also noticeable in heterotrophic protists and algae, in agreement with several studies that have detected a similar effect of river network position on eukaryotic communities (Wu and Liu 2018; Leira and Sabater 2005; Ortiz‐Vera et al. 2018; Guo et al. 2021; Zhang et al. 2023). Interestingly, this directional pattern was much less explored for several groups as fungi (Miura and Urabe 2015).

In relation to community diversity, LULC variables (forest area and extension of urban areas) explained a significant part of eukaryote diversity. It was noticeable that variables related to forest coverage (such as the area of broadleaf forests and silviculture plantations) had different effects reducing bacterial diversity while increasing the diversity of fungal, protistan and algal communities. Some authors reported lower bacterial richness in forested areas (Wood et al. 2017; Labouyrie et al. 2023) and argued that woodlands might be a highly competitive environment with reduced niche opportunities where fewer microbial taxa can persist. For instance, the Paiva catchment displayed low species richness of bacteria but the highest of algae and high dissimilarity compared to the other catchments. From all study cases, the Paiva catchment is the one with the highest coverage of eucalyptus plantations (sylviculture plantations), although the ecological status in most of the sampled stations is of excellent quality (Fonseca et al. 2020). With eucalyptus plantations dominating Paiva catchment, the lower bacterial diversity at Paiva could be due to the more recalcitrant character of eucalyptus leaves for decomposition in relation to native broadleaf species (Fabian et al. 2017).

Regarding community composition, it is worth mentioning that land use variables had a twofold and even threefold effect for bacteria and algae in comparison to fungi and protists. This effect consisted mainly on higher abundances for bacteria and algae involved in nitrogen cycling pathways. In our study, the catchments' water quality and quantity were impacted by agricultural practices that presumably affected nutrient loads, especially in the Couesnon River (Thomas et al. 2022). This effect also involved agricultural and urban areas in other catchments, such as the lower parts of the Miera river, Paiva or Carlingford Lough.

Finally, the changes observed in community composition in relation to river network position and land use types were also dependant on the higher scale environmental filters (climatic and geological characteristics). This is backed by the strong interactions found in this study between climatic‐geological variables and topographical and LULC in explaining community composition. These interactions show that global change effects on Atlantic RMC might be asymmetrical in relation to the region's climatic and geological characteristics.

#### 4.3.1 Potential Indicators of River Network Position and LULC

Our study shed evidence on RMC compositional changes along the river network and in relation to LULC characteristics.

In the middle or lower parts of the river network with a high percentage of limestone and lower average temperature during the year (Spanish and Carlingford Lough), we found an increasing abundance of Bacteroidetes such as *Fluviicola* spp. or *Flavobacterium* spp. These bacteria are common inhabitants of detrital aggregates, linked to algal bloom and the degradation of algal sulfated polysaccharides (Kosek et al. 2019). On the other hand, in the catchments characterised by higher average temperatures and a marked dry season (Paiva and Couesnon catchments), other Bacteroidetes dominate downstream (e.g., *Pseudarcicella* spp., NS11‐12_marine_group) as well as other bacteria from the Microbacteriaceae family. The changes in Bacteroidetes distribution found in this study agree with Niño‐García, Ruiz‐González, and Del Giorgio (2016), but they contrast with other studies which propose a Bacteroidetes decrease downstream (e.g., Read et al. 2015). Bacteroidetes are believed to degrade high‐molecular‐weight organic compounds, and therefore, we argue that downstream patterns could depend no only on river network position but also on the effect that land uses might have on the concentration of these organic compounds (Wang et al. 2017; Zhao et al. 2022).

Regarding eukaryotes, we found a higher relative abundance of the phylum Ascomycota in the midstream and downstream river reaches, which may be due to its critical role in degrading organic substrates (Chen et al. 2020) and also because of increased levels of nutrients, especially carbon and phosphorus (Chen et al. 2020; Guo et al. 2019). Ciliated protists and sessile ones whose part of their life cycle are free‐swimmers were also more prominent in downstream river reaches. In lower reaches we also found higher abundance of centric (*Cyclotella* sp., *Cyclostephanos* sp., or *Discostella* sp.) and motile diatoms (*Navicula* sp., or *Nitzschia* sp.). Centric diatoms have a planktonic life form and can dominate in eutrophic, turbid and slow‐flowing waters (Goldenberg‐Vilar et al. 2014; Muylaert and Sabbe 1999; Ha, Jang, and Joo 2003).

In the upper river reaches, the order Rhizobiales indicated forested streams although, interestingly, different taxa were characteristic of the warmer (Paiva and Couesnon) or the cooler and more humid catchments (northern Spanish and Carlingford Lough). Taxa from the phylum Basidiomycota (Agaricomycetes and Microbotryomycetes), which are mostly wood decayers (Hartmann et al. 2017), were also abundant in the upstream river reaches. Similarly, heterotrophic protists and algae were also found predominantly in upstream river reaches. These taxa involved the phylum Cercozoa (different taxa regarding the catchment) and algae from class Chrysophyceae (*Hydrurus* sp.) in the more humid catchments (Spanish and Carlingford Lough), while desmids (family Zygnematophyceae) were more abundant in the upstream sites of Paiva catchments.

Finally, the effect of agricultural and urban land uses was related to higher abundances of Gammaproteobacteria, such as *Malikia* spp. or *Rickettsiella* spp. *Malikia* spp. are associated to globally important nitrogen cycling pathways, including denitrification and nitrogen fixation, while *Rickettsiella* spp. is an animal pathogen (Yang et al. 2020). In addition, *Nitrospira* spp. was also positively associated to increases on agricultural and urban areas (e.g., Couesnon and Miera catchments). This bacteria is also involved in the nitrogen cycle (oxidising nitrite to nitrate; Daims and Wagner 2018) and is an indicator of high nitrogen concentration. In this regard, the Miera catchment is highly deforested with high nutrient levels in summer in its lower reaches due to reduced base flows (Belmar et al. 2018) and the effects of sewage effluents in the lower part of the catchment (Rodríguez‐Castillo et al. 2017). Another clear imprint of land use was the high occurrence of faecal indicators in the middle and lower reaches of the catchments with high pressure of livestock farming, especially the Spanish and Carlingford Lough catchments. The impacts of livestock farming were evident with a higher number of bacteria from the phylum Firmicutes, Bacteroidetes, Clostridia class and members from the family Ruminococcaceae, taxa prevalent in livestock‐affected and densely‐populated regions (Gao et al. 2022; Teixeira et al. 2020; Kosek et al. 2019).

## 5 Conclusions

This study has reported a massive invisible diversity of RMC across the Atlantic Europe, providing evidence of the role of different drivers of RMC diversity and composition. Diversity of prokaryotes were mostly affected by topographical and geological variables in comparison to the diversity of eukaryotic groups driven mainly by LULC.

On the other hand, species composition was highly consistent across taxa, with several of the same environmental factors being important for most groups. RMC compositional patterns denoted a strong regional footprint likely related to the environmental filter of climate and geology and secondly, to changes driven by variables operating at the catchment scale (topography and LULC). This delineates distinctions between communities in warmer catchments with dry seasons (Paiva and Couesnon catchments in France and Portugal) compared to those with cooler temperatures and more consistent year‐round rainfall (Spanish catchments and Carlingford Lough in the United Kingdom). Our research highlights varying sensitivities among the community composition of bacteria, fungi, heterotrophic protists and algae. Bacteria and fungal community composition exhibited more robust responses to climatic variables, contrasting with the higher sensitivity of heterotrophic protists and algae to geological characteristics (e.g., limestone or igneous rocks). Furthermore, the impact of land use variables was distinct, notably affecting the composition of bacteria and algae the most, especially regarding the percentage of agricultural areas, emphasising their involvement in nitrogen cycling pathways. Finally, the large extent of this study, together with a sampling scheme covering inter and intra‐catchment variability, has unveiled potential indicators of global change, including taxa favouring increasing temperatures, as well as indicators resilient to elevated nutrient levels in urban and more polluted agricultural areas. Microbes are at the basis of the food web and lead many key ecological functions, this study contributes to unmask RMC patterns in Atlantic catchments what could be paramount to address future actions to mitigate and adapt to the effects of global change.

This article directly contributes to several Sustainable Development Goals (SDGs), especially SDG 13 (Climate Action) and SDG 15 (Life on Land). By examining microbial biodiversity in river ecosystems and how these communities respond to climate change and land use alterations, the research addresses key aspects of climate resilience and ecosystem sustainability. Understanding microbial diversity's role in biogeochemical cycles and ecosystem functions is crucial for developing models to predict ecosystem responses to environmental changes which aligns with the goals of mitigating climate change and preserving biodiversity. Additionally, the study's focus on land use patterns, particularly agriculture and urbanisation, underscores its relevance to SDG 6 (Clean Water and Sanitation), as it enhances knowledge of water quality and ecosystem health, both essential for sustainable water management practices.

## Author Contributions

**Alejandra Goldenberg‐Vilar:** conceptualization, investigation, writing – original draft, methodology, visualization, writing – review and editing, software, formal analysis, data curation. **María Morán‐Luis:** conceptualization, investigation, writing – original draft, methodology, visualization, software, formal analysis, data curation. **David R. Vieites:** investigation, writing – review and editing, methodology. **José Manuel Álvarez‐Martínez:** investigation, writing – review and editing, software, methodology. **Ana Silió:** investigation, methodology, writing – review and editing, software. **Cendrine Mony:** investigation, writing – review and editing. **Simone Varandas:** investigation. **Sandra Mariza Monteiro:** investigation. **Diane Burgess:** investigation. **Edna Cabecinha:** investigation, writing – review and editing, funding acquisition. **José Barquín:** conceptualization, investigation, funding acquisition, writing – review and editing, project administration, supervision.

## Conflicts of Interest

The authors declare no conflicts of interest.

## Supporting information


Data S1.

## Data Availability

The data that support the findings of this study are available on request from the corresponding author.
