# Supplementary material for: Biogeographical Distribution of River Microbial Communities in Atlantic Catchments
Source: Environ Microbiol Rep. 2025 Jan 8;17(1):e70065. doi: 10.1111/1758-2229.70065 (PMC11707552; doi:10.1111/1758-2229.70065)
Supplement: Supplementary file 1 — Data S1. [file EMI4-17-e70065-s001.docx]

**Supplementary material**

Table S1. Description of environmental variables and its source


Table S2. Spatial data sources per country

Table S3. Average values of environmental variables per catchment. See Table S1 for environmental variable codes.

| **VARIABLE** | **Carlingford-Lough** | **Couesnon** | **Ason** | **Miera** | **Pas** | **Paiva** |
| --- | --- | --- | --- | --- | --- | --- |
| AREA_SQKM | 47.75 | 171.66 | 140.82 | 109.99 | 101.70 | 193.67 |
| ELEV_M | 18.11 | 39.17 | 139.25 | 154.54 | 206.56 | 395.96 |
| OUT_DIST | 26.99 | 41.36 | 17.81 | 20.64 | 32.83 | 53.38 |
| STRM_ORDER | 3.23 | 4.14 | 5.16 | 4.93 | 4.95 | 3.94 |
| WIDTH_m | 8.92 | 10.25 | 13.10 | 13.32 | 12.94 | 16.24 |
| FLOW_ | 0.63 | 0.54 | 1.84 | 0.80 | 0.47 | 1.25 |
| FRE3 | 4.38 | 4.89 | 7.05 | 7.73 | 5.81 | 4.17 |
| TMESU | 11.38 | 16.39 | 13.12 | 13.67 | 12.75 | 17.19 |
| MN_blf | 0.02 | 0.14 | 0.11 | 0.08 | 0.13 | 0.21 |
| BF_blf | 0.02 | 0.23 | 0.34 | 0.29 | 0.14 | 0.47 |
| MN_agr | 0.02 | 0.41 | 0.01 | 0.02 | 0.01 | 0.14 |
| BF_agr | 0.04 | 0.10 | 0.01 | 0.03 | 0.01 | 0.17 |
| MN_uhd | 0.03 | 0.08 | 0.01 | 0.02 | 0.01 | 0.03 |
| BF_uhd | 0.23 | 0.09 | 0.06 | 0.11 | 0.06 | 0.02 |
| MN_gepdelm | 0.01 | 0.00 | 0.15 | 0.18 | 0.15 | 0.02 |
| BF_gepdelm | 0.00 | 0.00 | 0.09 | 0.08 | 0.11 | 0.05 |
| MN_IG | 0.50 | 0.00 | 0.00 | 0.00 | 0.02 | 0.70 |
| MN_LM | 0.00 | 0.00 | 0.33 | 0.39 | 0.15 | 0.00 |
| MN_SD | 0.50 | 0.00 | 0.59 | 0.46 | 0.29 | 0.30 |
| MN_SQ | 0.00 | 0.00 | 0.67 | 0.15 | 0.41 | 0.00 |
| MN_syl | 0.05 | 0.04 | 0.12 | 0.05 | 0.04 | 0.27 |
| BF_syl | 0.00 | 0.02 | 0.03 | 0.03 | 0.08 | 0.22 |
| MN_tx | 8.46 | 15.19 | 15.92 | 16.18 | 15.63 | 18.87 |
| MN_tn | 3.70 | 7.55 | 5.27 | 5.76 | 4.93 | 7.48 |
| MN_tg | 6.06 | 11.34 | 8.23 | 8.92 | 7.73 | 13.05 |
| MN_rr | 998.75 | 696.69 | 1522.60 | 1713.87 | 1557.84 | 1352.64 |

Table S4. ANOVA and Tukey post-hoc tests for the comparison of rarefied richness between catchments. Only the results of the groups that yield significant results (Prokaryotes and algae) are shown. N.s. Not significant.

|  | **Prokaryotes** | **Algae** |
| --- | --- | --- |
|  | Pr(>F) | Pr(>F) |
| ANOVA overall significance | 0.0019 | 0.003 |
|  |  |  |
| Tukey post-hoc | p adjusted | p adjusted |
| Paiva-Ason | n.s. | 0.002 |
| Paiva-Miera | 0.002 | 0.05 |
| Paiva-Pas | 0.02 | 0.01 |
| Couesnon-Paiva | n.s | 0.03 |

Table S5. Results of the pairwise –adonis test on differences between RMC communities for all catchment comparisons per taxonomic group (Prokaryotes, fungi, protist and algae). F-test statistics and adjusted p values are shown. P-adjusted values for not significant results are left blank.

|  | **Prokaryotes** | | **Fungi** | | **Protist** | | **Algae** | |
| --- | --- | --- | --- | --- | --- | --- | --- | --- |
| Site comparison | F-Model | p-adjusted | F-Model | p-adjusted | F-Model | p-adjusted | F-Model | p-adjusted |
| Asón - Carlingford | 3.39 |  | 3.48 | 0.015 | 3.28 | 0.015 | 5.30 | 0.015 |
| Asón - Couesnon | 9.62 | 0.015 | 10.56 | 0.015 | 4.31 | 0.015 | 11.75 | 0.015 |
| Asón - Miera | 2.53 |  | 1.42 |  | 1.02 |  | 1.46 |  |
| Asón - Paiva | 12.7 | 0.015 | 13.89 | 0.015 | 8.32 | 0.015 | 13.66 | 0.015 |
| Asón - Pas | 3.95 |  | 2.86 | 0.03 | 1.33 | 0.03 | 2.05 |  |
| Carlingford - Couesnon | 6.14 | 0.015 | 5.32 | 0.015 | 3.29 | 0.015 | 4.91 | 0.015 |
| Carlingford - Miera | 2.74 |  | 2.75 | 0.03 | 2.86 | 0.03 | 3.88 | 0.03 |
| Carlingford - Paiva | 9.86 | 0.015 | 7.63 | 0.015 | 2.95 | 0.015 | 7.36 | 0.015 |
| Carlingford - Pas | 2.38 |  | 3.04 |  | 2.34 |  | 3.35 | 0.045 |
| Couesnon - Miera | 8.52 | 0.015 | 8.52 | 0.015 | 3.65 | 0.015 | 8.72 | 0.015 |
| Couesnon - Paiva | 12.46 | 0.015 | 8.67 | 0.015 | 5.16 | 0.015 | 10.55 | 0.015 |
| Couesnon - Pas | 9.95 | 0.015 | 8.02 | 0.015 | 4.29 | 0.015 | 10.48 | 0.015 |
| Miera - Paiva | 12.52 | 0.015 | 10.33 | 0.015 | 7.47 | 0.015 | 11.59 | 0.015 |
| Miera - Pas | 1.96 |  | 1.38 |  | 1.05 |  | 1.93 |  |
| Paiva - Pas | 12.83 | 0.015 | 9.04 | 0.015 | 6.51 | 0.015 | 10.50 | 0.015 |

Table S6. Variation partitioning results for RMC richness showing adjusted variation, percentage of explained variation, percentage explained from total variation and degrees of freedom for each fraction and combined fractions. Clim-geo: climatic and geological variables; Topo: Topographical variables; LULC: Land use and land cover variables.

| **Prokaryotes** |  |  |  |  |
| --- | --- | --- | --- | --- |
| **Fraction** | **Variation(adj)** | **% of Explained** | **% of All** | **DF** |
| Clim-geo | 0.23 | 57.63 | 23.405 | 5 |
| Topo | 0.12 | 30.22 | 12.272 | 2 |
| LULC | 0.04 | 8.84 | 3.589 | 2 |
| Clim-geo + Topo | -0.04 | -- | -3.793 | -- |
| Topo + LULC | 0.01 | 1.48 | 0.603 | -- |
| Clim-geo + LULC | 0.01 | 1.29 | 0.523 | -- |
| Shared by all | 0.00 | 0.55 | 0.222 | -- |
| Total Explained | 0.37 | 100.00 | 36.82 | 9.00 |
|  |  |  |  |  |
| **Fungi** |  |  |  |  |
| **Fraction** | **Variation(adj)** | **% of Explained** | **% of All** | **DF** |
| Clim-geo | 0.07381 | 31.20 | 7.381 | 4 |
| Topo | 0.01716 | 7.25 | 1.716 | 1 |
| LULC | 0.13847 | 58.53 | 13.847 | 4 |
| Clim-geo + Topo | -0.0004 | -- | -0.04 | -- |
| Topo + LULC | 0.00713 | 3.01 | 0.713 | -- |
| Clim-geo + LULC | -0.05461 | -- | -5.461 | -- |
| Shared by all | -0.0067 | -- | -0.67 | -- |
| Total Explained | 0.17486 | 100.00 | 17.486 | 9 |
|  |  |  |  |  |
| **Protist** |  |  |  |  |
| **Fraction** | **Variation(adj)** | **% of Explained** | **% of All** | **DF** |
| Clim-geo | 0.0755 | 37.16 | 7.55 | 3 |
| Topo | -- | 0.00 | -- |  |
| LULC | 0.12768 | 62.84 | 12.768 | 3 |
| Clim-geo + Topo | -- | 0.00 | -- | -- |
| Topo + LULC | -- | 0.00 | -- | -- |
| Clim-geo + LULC | -0.0315 | -- | -3.15 | 6 |
| Shared by all | -- | 0.00 | -- | -- |
| Total Explained | 0.17168 | 100.00 | 17.168 | 12 |
|  |  |  |  |  |
| **Algae** |  |  |  |  |
| **Fraction** | **Variation(adj)** | **% of Explained** | **% of All** | **DF** |
| Clim-geo | 0.12641 | 46.28 | 12.641 | 1 |
| Topo | -- | 0.00 | -- | -- |
| LULC | 0.14674 | 53.72 | 14.674 | 4 |
| Clim-geo + Topo | -- | 0.00 | -- | -- |
| Topo + LULC | -- | 0.00 | -- | -- |
| Clim-geo + LULC | -0.05327 | -- | -5.327 | -- |
| Shared by all | -- | 0.00 | -- | -- |
| Total Explained | 0.21988 | 100.00 | 21.988 | 5 |

Table S7. Variation partitioning results for RMC composition based on RDA analysis showing adjusted variation, percentage of explained variation, percentage explained from total variation, degrees of freedom and mean squares for each fraction and combined fractions. Clim-geo: climatic and geological variables; Topo: Topographical variables; LULC: Land use and land cover variables.

| **Prokaryotes** |  |  |  |  |  |
| --- | --- | --- | --- | --- | --- |
| **Fraction** | **Variation(adj)** | **% of Explained** | **% of All** | **DF** | **Mean Square** |
| Clim-geo | 0.085686 | 24.6 | 8.6 | 5 | 0.023 |
| Topo | 0.054965 | 15.8 | 5.5 | 3 | 0.023 |
| LULC | 0.02941 | 8.4 | 2.9 | 4 | 0.013 |
| Clim-geo + Topo | 0.05304 | 15.2 | 5.3 | -- | -- |
| Topo + LULC | 0.0097434 | 2.8 | 1 | -- | -- |
| Clim-geo + LULC | 0.062378 | 17.9 | 6.2 | -- | -- |
| Shared by all | 0.05346 | 15.3 | 5.3 | -- | -- |
| Total Explained | 0.34868 | 100 | **34.9** | 12 | 0.036 |
|  |  |  |  |  |  |
| **Fungi** |  |  |  |  |  |
| **Fraction** | **Variation(adj)** | **% of Explained** | **% of All** | **DF** | **Mean Square** |
| Clim-geo | 0.055675 | 22.7 | 5.6 | 6 | 0.016 |
| Topo | 0.041739 | 17.1 | 4.2 | 4 | 0.017 |
| LULC | 0.0091882 | 3.8 | 0.9 | 5 | 0.009 |
| Clim-geo + Topo | 0.03265 | 13.3 | 3.3 | -- | -- |
| Topo + LULC | -0.0019585 | -0.8 | -0.2 | -- | -- |
| Clim-geo + LULC | 0.043157 | 17.6 | 4.3 | -- | -- |
| Shared by all | 0.064319 | 26.3 | 6.4 | -- | -- |
| Total Explained | 0.24477 | 100 | **24.5** | 15 | 0.024 |
|  |  |  |  |  |  |
| **Protist** |  |  |  |  |  |
| **Fraction** | **Variation(adj)** | **% of Explained** | **% of All** | **DF** | **Mean Square** |
| Clim-geo | 0.063181 | 34.8 | 6.3 | 5 | 0.02 |
| Topo | 0.025702 | 14.2 | 2.6 | 4 | 0.014 |
| LULC | 0.0045809 | 2.5 | 0.5 | 5 | 0.009 |
| Clim-geo + Topo | 0.0048136 | 2.7 | 0.5 | -- | -- |
| Topo + LULC | 0.0061564 | 3.4 | 0.6 | -- | -- |
| Clim-geo + LULC | 0.040642 | 22.4 | 4.1 | -- | -- |
| Shared by all | 0.036265 | 20 | 3.6 | -- | -- |
| Total Explained | 0.18134 | 100 | **18.1** | 14 | 0.021 |
|  |  |  |  |  |  |
| **Algae** |  |  |  |  |  |
| **Fraction** | **Variation(adj)** | **% of Explained** | **% of All** | **DF** | **Mean Square** |
| Clim-geo | 0.10006 | 35.4 | 10 | 5 | 0.026 |
| Topo | 0.035848 | 12.7 | 3.6 | 3 | 0.018 |
| LULC | 0.02379 | 8.4 | 2.4 | 5 | 0.012 |
| Clim-geo + Topo | 0.0050555 | 1.8 | 0.5 | -- | 3 |
| Topo + LULC | 0.0067241 | 2.4 | 0.7 | -- | 5 |
| Clim-geo + LULC | 0.07055 | 24.9 | 7.1 | -- | 4 |
| Shared by all | 0.040859 | 14.4 | 4.1 | -- | 6 |
| Total Explained | 0.28288 | 100 | **28.3** | 13 | 0.029 |

Table S8. Percentage of explained variation, contribution and statistics of each fraction separately and por taxonomical group based on RDA analysis. See Table S1 for environmental variable codes.

Table S9. Richness models coefficients and derived statistics for each taxonomical group.

| Prokaryote model Coefficients | | | | |
| --- | --- | --- | --- | --- |
|  | Estimate | Std. Error | t value | Pr(>\|t\|) |
| (Intercept) | 13125.16 | 1251.6 | 10.487 | < 2e-16 *** |
| AREA_SQKM | -864.27 | 245.57 | -3.519 | 0.000691 *** |
| FRE3 | 337 | 131.43 | 2.564 | 0.012058 * |
| MN_uhd | -15744.02 | 8465.87 | -1.86 | 0.066307 . |
| MN_IG | -2205.68 | 611.27 | -3.608 | 0.000514 *** |
| MN_SD | -7014.69 | 1930.88 | -3.633 | 0.000473 *** |
| MN_SQ | -1396.75 | 603.84 | -2.313 | 0.023075 * |
| MN_syl | 8656.07 | 4500.84 | 1.923 | 0.057724 . |
| MN_tg | -147.63 | 86.86 | -1.7 | 0.092763 . |
| ELEV_M | 393.86 | 270.92 | 1.454 | 0.149596 |
|  |  |  |  |  |
| Fungi model Coefficients | | | | |
|  | Estimate | Std. Error | t value | Pr(>\|t\|) |
| (Intercept) | 793.02 | 86.24 | 9.195 | 1.79e-14 *** |
| AREA_SQKM | 59.92 | 35.62 | 1.682 | 0.09609 . |
| MN_blf | 1087.29 | 697.74 | 1.558 | 0.1228 |
| BF_blf | 367.29 | 233.98 | 1.57 | 0.1201 |
| BF_agr | 228.29 | 148.64 | 1.536 | 0.12821 |
| BF_gepdelm | 2994.23 | 948.4 | 3.157 | 0.00219 ** |
| MN_IG | -115.47 | 81.69 | -1.413 | 0.1611 |
| MN_LM | -159.06 | 99.54 | -1.598 | 0.11366 |
| MN_SD | 606.77 | 265.53 | 2.285 | 0.02473 * |
| MN_SQ | -225.42 | 88.22 | -2.555 | 0.01235 * |
|  |  |  |  |  |
| Protist model Coefficients | | | | |
|  | Estimate | Std. Error | t value | Pr(>\|t\|) |
| (Intercept) | 7.84E+02 | 7.47E+01 | 10.487 | < 2e-16 *** |
| BF_blf | 4.25E+02 | 1.72E+02 | 2.464 | 0.015633 * |
| MN_uhd | -1.79E+03 | 1.00E+03 | -1.79 | 0.076766 . |
| BF_gepdelm | 2.72E+03 | 7.88E+02 | 3.454 | 0.000844 *** |
| MN_SQ | -1.92E+02 | 6.43E+01 | -2.984 | 0.003667 ** |
| MN_SD | 4.13E+02 | 2.17E+02 | 1.906 | 0.059848 . |
| MN_rr | -7.85E-02 | 5.71E-02 | -1.375 | 0.17266 |
|  |  |  |  |  |
| Algae model Coefficients | | | | |
|  | Estimate | Std. Error | t value | Pr(>\|t\|) |
| (Intercept) | 1061.33 | 52.83 | 20.089 | < 2e-16 *** |
| BF_blf | 568.53 | 269.14 | 2.112 | 0.037393 * |
| MN_uhd | -2223.81 | 1410.9 | -1.576 | 0.118458 |
| BF_gepdelm | 2057.5 | 1148.78 | 1.791 | 0.076612 . |
| MN_SQ | -397.47 | 99.66 | -3.988 | 0.000134 *** |
| MN_syl | 1698.63 | 582.88 | 2.914 | 0.004488 ** |
